# Muscle and reflex changes with varying joint angle in hemiparetic stroke

**DOI:** 10.1186/1743-0003-5-6

**Published:** 2008-02-27

**Authors:** Mehdi M Mirbagheri, Laila Alibiglou, Montakan Thajchayapong, William Z Rymer

**Affiliations:** 1 Sensory Motor Performance Program, Rehabilitation Institute of Chicago, Chicago, USA; 2 Department of Physical Medicine and Rehabilitation, Northwestern University, Chicago, USA; 3 Interdepartmental Neuroscience Program, Northwestern University, Chicago, USA; 4 Department of Mechanical Engineering, Northwestern University, Chicago, USA

## Abstract

**Background:**

Despite intensive investigation, the origins of the neuromuscular abnormalities associated with spasticity are not well understood. In particular, the mechanical properties induced by stretch reflex activity have been especially difficult to study because of a lack of accurate tools separating reflex torque from torque generated by musculo-tendinous structures. The present study addresses this deficit by characterizing the contribution of neural and muscular components to the abnormally high stiffness of the spastic joint.

**Methods:**

Using system identification techniques, we characterized the neuromuscular abnormalities associated with spasticity of ankle muscles in chronic hemiparetic stroke survivors. In particular, we systematically tracked changes in muscle mechanical properties and in stretch reflex activity during changes in ankle joint angle. Modulation of mechanical properties was assessed by applying perturbations at different initial angles, over the entire range of motion (ROM). Experiments were performed on both paretic and non-paretic sides of stroke survivors, and in healthy controls.

**Results:**

Both reflex and intrinsic muscle stiffnesses were significantly greater in the spastic/paretic ankle than on the non-paretic side, and these changes were strongly position dependent. The major reflex contributions were observed over the central portion of the angular range, while the intrinsic contributions were most pronounced with the ankle in the dorsiflexed position.

**Conclusion:**

In spastic ankle muscles, the abnormalities in intrinsic and reflex components of joint torque varied systematically with changing position over the full angular range of motion, indicating that clinical perceptions of increased tone may have quite different origins depending upon the angle where the tests are initiated.

Furthermore, reflex stiffness was considerably larger in the non-paretic limb of stroke patients than in healthy control subjects, suggesting that the non-paretic limb may not be a suitable control for studying neuromuscular properties of the ankle joint.

Our findings will help elucidate the origins of the neuromuscular abnormalities associated with stroke-induced spasticity.

## Introduction

Injury to the central nervous system, as occurs in stroke, results in several forms of motor and/or sensory impairment including spasticity, a hallmark of the upper motoneuron syndrome [1-7]. A widely accepted definition of spasticity, offered by Lance, describes spasticity as a velocity-dependent joint resistance to stretch [8]. Most scientific studies have focused on neural mechanisms because the primary lesion causing spasticity is located in the central nervous system. In recent years, there have been reports that attribute the increased joint resistance to structural and mechanical changes in skeletal muscles [9-12]. Thus, despite decades of extensive research, the relative contributions of reflex mechanisms and of changes in muscular and connective tissues remain unclear.

Changes in neuromuscular properties can be well characterized by measuring joint dynamic stiffness, which is the dynamic relationship between joint angular perturbation as input and the resulting torque as output [13,14]. Joint dynamic stiffness is determined by both intrinsic and reflex mechanisms. Intrinsic stiffness arises from muscle fibers, and from surrounding connective tissues, whereas reflex stiffness arises from the neural response to muscle stretch. These mechanisms coexist, are interdependent, and can change dramatically over time. Since the mechanical contributions of these various sources of stiffness vary under different functional conditions such as joint position and voluntary contraction levels [11,14], it is often difficult to separate them, and consequently to fully characterize the mechanical joint behavior [15]. This explains why several attempts have been undertaken to separate intrinsic and reflex torque and/or stiffness using electrical stimulation [16-18] and nerve block [19] to suppress the reflex response.

These experimental approaches have met with limited success as described in detail in our previous publications [11,14].

To explore the limitations of previous analytical approaches briefly, in some cases sinusoidal inputs were applied and Fourier analysis used to extract the component of the output at the input frequency and all other components discarded [20-23]. This analysis procedure explicitly excludes nonlinear contributions to joint dynamic stiffness, and would ignore almost all of the reflex torque. Other studies have used indirect analyses to relate the "path-length" of the Nyquist diagram to reflex stiffness [20-23]. This method also assumes a linear model, whereas reflex stiffness is strongly non-linear even for small perturbations about an operating point [13,14,24]. Consequently, the path-length approach is likely to provide inaccurate estimates of reflex contributions to overall stiffness.

To address some of these limitations, we have developed a parallel cascade system identification technique [13,14] to characterize joint dynamic stiffness and to separate its intrinsic and reflex components. In our published studies of spinal cord injured persons using this technique, we reported that overall ankle dynamic stiffness was abnormally high. Both intrinsic and reflex mechanical responses were significantly increased, but the major mechanical abnormality arose from increased reflex stiffness [11,25]. In contrast, Galiana et al. reported no significant difference in intrinsic stiffness of the ankle joint in stroke subjects [26]. They also found that reflex stiffness increased only in a minority of their subjects and was in a normal range overall, as has also been reported by Sinkjaer et al. [12].

The results of the Galiana et al. study showed that the ankle range of motion (ROM) of their subjects was limited, and extended only to the neutral position (90°), whereas our previous results indicated that the abnormalities were manifested mostly at mid-range and beyond, especially at full-dorsiflexion (DF) [11]. Thus, it is not surprising that they did not observe significant changes in the mechanical properties of the spastic ankle in stroke survivors. Sinkjaer et al. also measured reflex torque in response to a 4° stretch at a single position, however this test did not detect abnormalities in reflex mechanical properties.

On the other hand, it is also possible that the nature and origin of spasticity are different in various neurological disorders, such as between stroke and spinal cord injury. Thus, the contributions of different neuromuscular components to the spastic joint in the stroke population have not been sufficiently investigated. This study addressed these issues by examining the modulation of the abnormalities in intrinsic and reflex stiffness with changing ankle joint angle over the complete range of available angular motion in chronic, spastic stroke patients and in normal subjects.

Our findings are that both intrinsic and reflex stiffness increase abnormally in the spastic limb and that both series of changes are strongly, but differently, position dependent.

These findings are quite consistent with earlier published findings obtained in subject with spinal cord injury (SCI) [11,25], suggesting that although the cause and location of injury are different in spastic stroke and SCI subjects, the mechanical abnormalities were similar in most subjects in the two groups.

## Methods

### Subjects

Twenty individuals with a single hemispheric stroke (59.2 ± 9.9 years), and eleven age-matched healthy subjects (52.8 ± 10.9 years) participated in this study. All stroke survivors had chronic stroke of between 2 and 18 years (7.7 ± 4.4 years) duration, with different degrees of clinically assessed spasticity. Both paretic and non-paretic sides of the stroke subjects were tested. The healthy subjects were used as an additional control.

Patients met the following inclusion criteria: stable medical condition, absence of aphasia or significant cognitive impairment, absence of muscle tone abnormalities and motor or sensory deficits in the non-paretic leg, absence of severe muscle wasting or overt sensory deficits in the paretic lower limb, and spasticity in the involved ankle muscles for a duration of at least 1 year.

All subjects gave informed consent to the experimental procedures, which had been reviewed and approved by Northwestern University Institutional Review Board (IRB) Board.

### Clinical assessment

All stroke subjects were evaluated clinically using the Modified 6-point Ashworth Scale (MAS) to assess spasticity [27,28]. The MAS is a conventional clinical measure of spasticity.

The experiment was carried out on both paretic and non-paretic ankle joints. Although the *non-paretic *limb may sometimes have minor detectable impairments [29], it was designated as a control for the impaired limb because it is not spastic and has similar musculo-tendon architecture and limb mass. However, to control for possible changes in the non-paretic side, we used healthy age-matched subjects as additional controls.

### Apparatus

**Figure 1:**
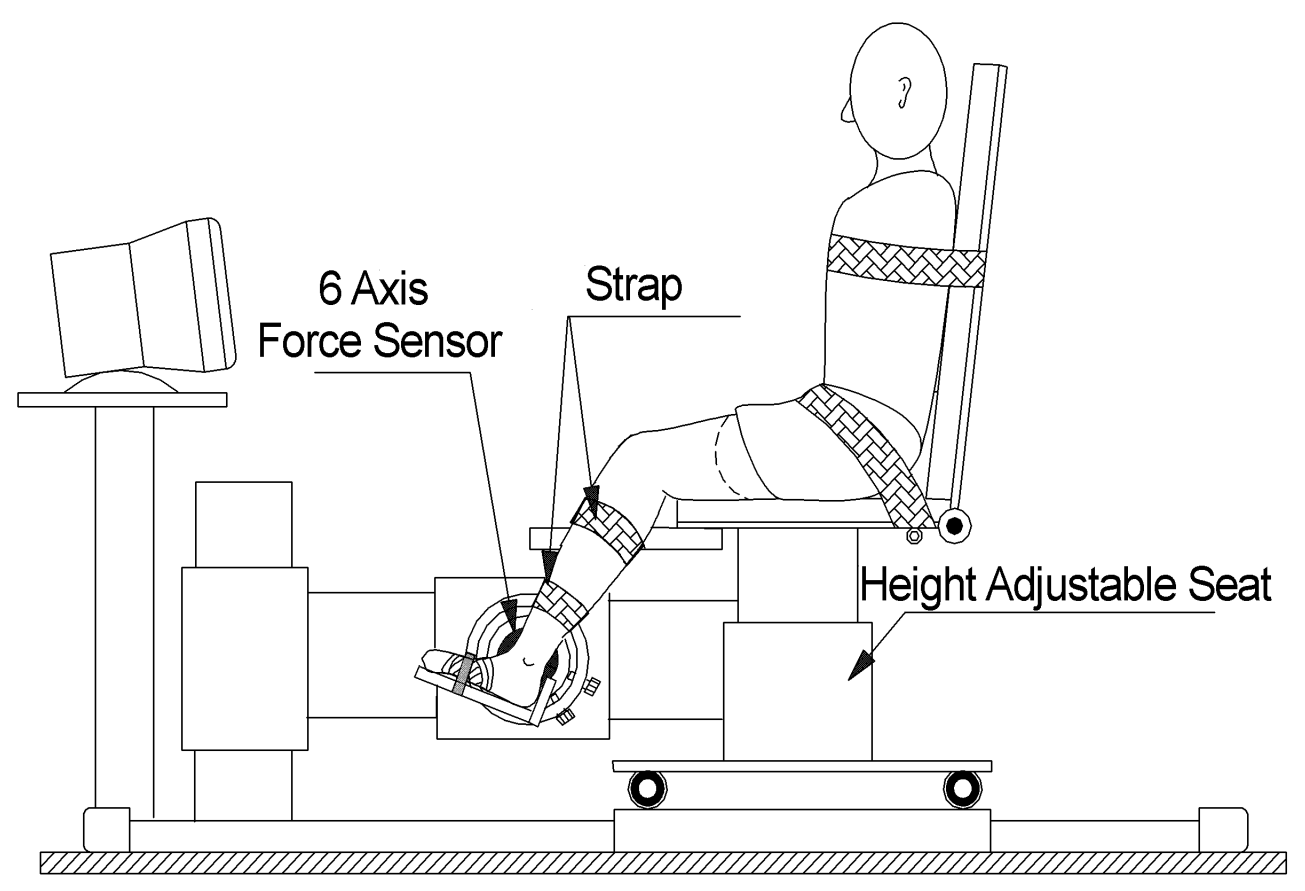
The apparatus including the joint stretching motor device, the height adjustable chair, and force and position sensors.

The joint stretching motor device operated as a position control servo driving ankle position to follow a command input (Figure 1). Subjects were seated and secured in an adjustable, chair with the ankle strapped to the footrest and the thigh and trunk strapped to the chair.

The seat and footrest were adjusted to align the ankle axis of rotation with the axis of the force sensor and the motor shaft. An oscilloscope mounted in front of the subject displayed a target signal and provided feedback of low-pass filtered joint torque.

### Recording

Ankle position was measured with a precision potentiometer. Torque was recorded using a 6-axis torque transducer, mounted between the beam of the footrest and the motor shaft. Displacements in the plantarflexion (PF) direction were taken as negative and those in the dorsiflexion (DF) direction as positive. An ankle angle of 90 degrees was considered to be the Neutral Position (NP) and defined as zero. Torque was assigned a polarity consistent with the direction of the movement that it would generate (e.g. DF torque was taken as positive). Electromyograms (EMGs) from tibialis anterior (TA) and lateral gastrocnemius (GS) were recorded using bipolar surface electrodes (Delsys, Inc. Boston, MA). Position, torque, and EMGs were filtered at 230 Hz to prevent aliasing, and sampled at 1 kHz by a 16 bit A/D.

### Procedures

#### Range of motion (ROM)

ROM was determined with the subject's ankle attached to the motor and manually moved to maximum PF and DF. Mean displacement amplitude was assessed 3 times by slowly moving the joint until the examiner perceived rapidly increasing resistance or the subject reported discomfort. The typical angular range was from about 50° PF (mean 49° ± 6°SD) to 20° DF (mean 21° ± 5° SD).

#### Paradigm

To identify overall stiffness properties and to separate the reflex and intrinsic components, we used Pseudorandom Binary Sequence (PRBS) position inputs with amplitude of 0.03 rad and a switching interval of 150 ms. Our previously published results demonstrated that these perturbations have a mean velocity low enough to avoid attenuating reflex responses, contain power over a wide enough bandwidth to identify the dynamics, and are well tolerated by the spastic subjects [30].

Trials were conducted at different ankle positions from full-PF to full-DF, at 5 degree intervals. Each position was examined under *Passive *conditions, where subjects were instructed to remain relaxed.

Following each trial, the torque and EMG signals were examined for evidence of non-stationarities or co-activation of TA. If there was evidence of either, the data were discarded and the trial was repeated.

### Analysis procedures

#### Parallel cascade identification technique

**Figure 2:**
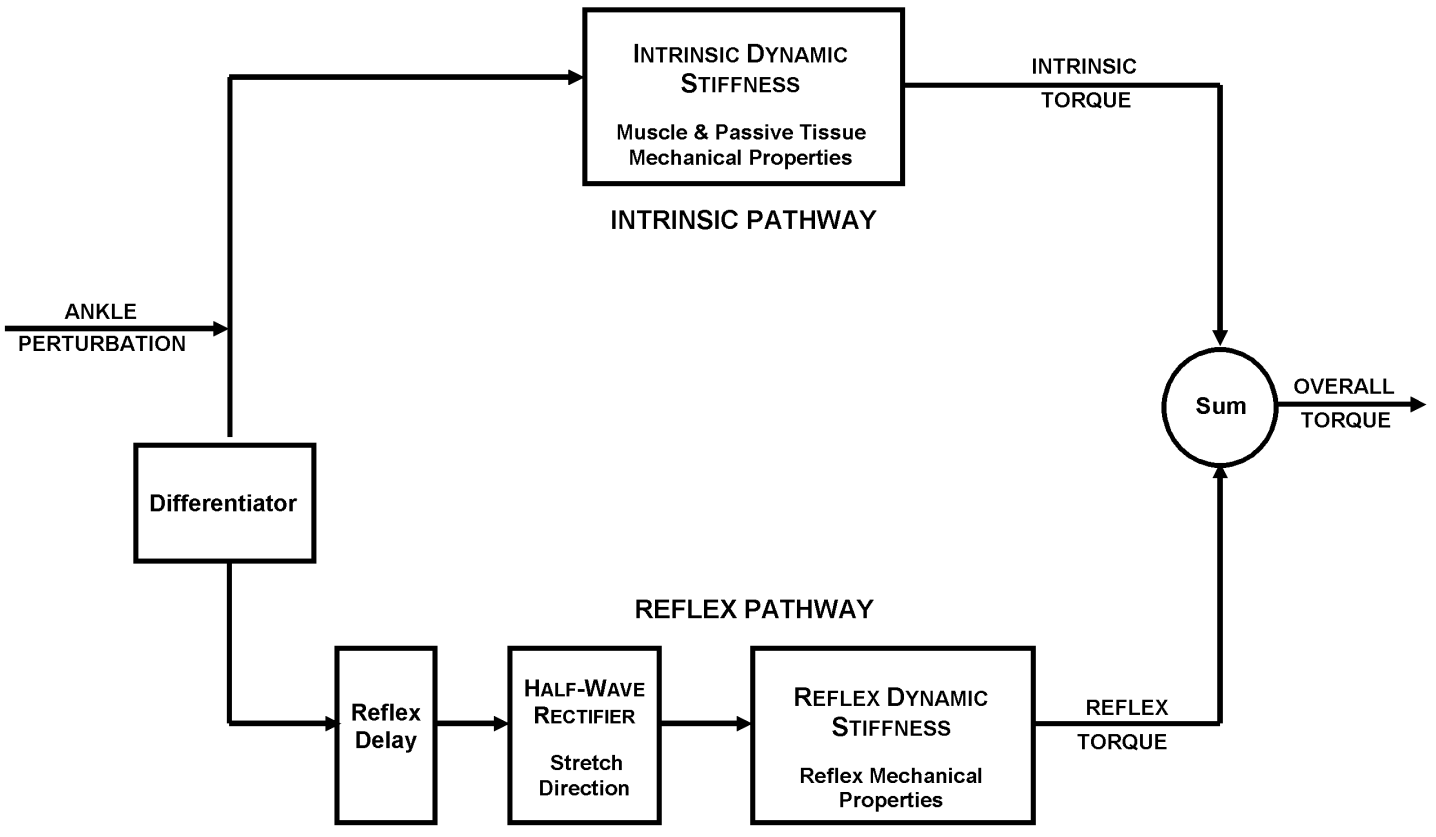
The parallel cascade structure used to identify intrinsic and reflex stiffness. Intrinsic dynamic stiffness is represented in the upper pathway by the intrinsic stiffness impulse response function. Reflex dynamic stiffness is represented by the lower pathway as a differentiator, followed by a static nonlinear element and then a linear impulse response function. The nonlinear element is a half wave rectifier which shows the direction of stretch.

Dynamic stiffness of the ankle is defined as the dynamic relation between joint position (as input), and resulting torque (as output). Reflex and intrinsic contributions to ankle dynamic stiffness were identified using a parallel cascade technique, described in detail in earlier publications [13,14]. Briefly, the method proceeded as shown in Figure 2.

Intrinsic stiffness dynamics (top pathway) were estimated in terms of a linear Impulse Response Function (IRF) relating position and torque. The reflex pathway (bottom pathway) was modeled as a differentiator in series with a delay, a static non-linear element (closely resembling a half-wave rectifier), and a dynamic linear element. Reflex stiffness dynamics were estimated by determining the IRF between half-waved rectified velocity as the input and reflex torque as the output. The intrinsic and reflex stiffness IRFs were convolved with the experimental input to predict the intrinsic and reflex torque respectively.

Linear models were fitted to the estimated intrinsic and reflex IRF curves using the Levenberg Marquardt nonlinear least-square fit algorithm [31]. To make fitting easier, the intrinsic stiffness IRF was inverted to give a compliance IRF, which was described by a second-order model having inertia, viscous and elastic parameters [14]. The intrinsic elastic parameter also corresponds to the steady-state, intrinsic stiffness gain.

The reflex stiffness was described by reflex delay and a third-order model having gain, damping, and frequency parameters. This model is more complex than the second-order model used in our previous work [13]. This is because we found that an additional pole was required to accurately fit the reflex IRFs of the spastic joint [32].

#### Statistical analysis

We used a two-way ANOVA test, and standard t-tests to analyze our results. Two-way ANOVA analyses were used to test for significant main effects due to subject groups, joint positions, or their interactions. The results could tell us if there were significant differences due to main effects and/or their interactions. Tukey post-hoc comparisons were performed to find at which positions the differences between groups were significant.

Standard t-tests procedures were used to test for significant changes in intercepts and slopes of reflex stiffness as a function of joint angle.

Results with *p *values less than 0.05 were considered significant.

## Results

### Experimental data

To illustrate the form of data that are collected in our experiments, we present a sequence of typical experimental records, together with results of model predictions.

**Figure 3:**
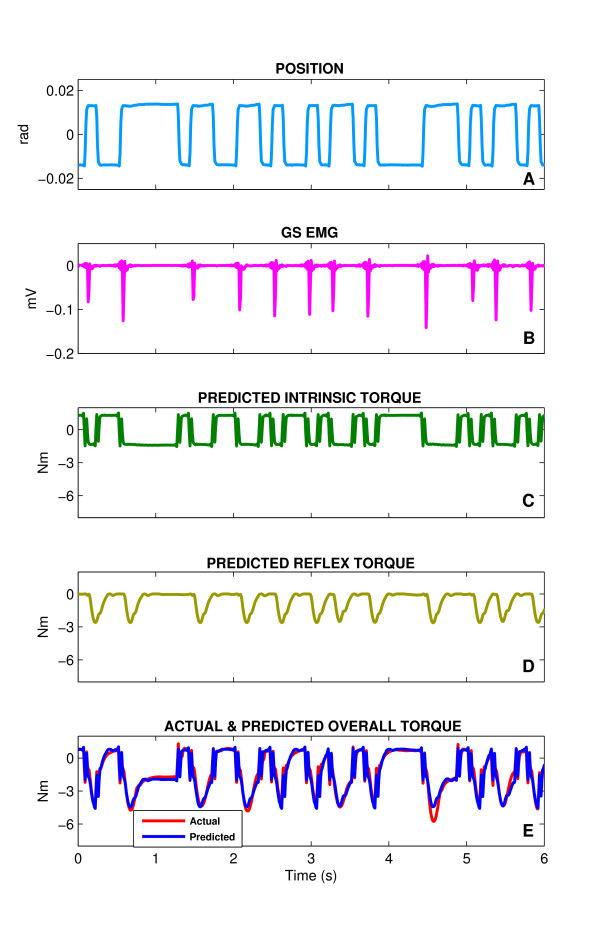
A segment from a typical sequence trial for a spastic under relaxed conditions. **A **Position, **B **Half-wave rectified gastrocnemius electromyogram (GS), **C **Predicted intrinsic torque, **D **Predicted reflex torque and **E **Predicted overall torque (thick curve) superimposed on the actual torque (thin curve). Displacements in the PF direction were taken as negative and those in the DF direction as positive. Torque was assigned a polarity consistent with the direction of the movement that it would generate (e.g. PF torque was taken as negative).

Figure 3 shows a segment of a typical PRBS trial with the amplitude of 0.03 rad and the switching-rate of 150 ms. This record was acquired while the subject was relaxed. Angular displacements in the positive (dorsiflexing) direction (Fig. 3A) evoked a short latency burst of activity in gastrocnemius (GS) (Fig. 3B) while displacements in the negative (plantarflexing) direction evoked no response. The torque record (Fig. 3E) is similarly asymmetric, in that dorsiflexing displacements evoked torque responses having intrinsic and reflex components, while responses to plantarflexing displacements have only the intrinsic component. The intrinsic and reflex torque predicted by the parallel-cascade identification model are shown in Fig. 3C and Fig. 3D, respectively. The model's estimate of the overall torque, given by the sum of the intrinsic and reflex torques, is shown in thick curve superimposed on the experimentally observed torque shown in thin curve (Fig. 3E). It is evident that the overall prediction was very good; in this case, it accounted for 92.2% of the observed torque variance. This was typical of all our data; the parallel-cascade model routinely accounted for more than 90% of the overall torque variance.

**Figure 4:**
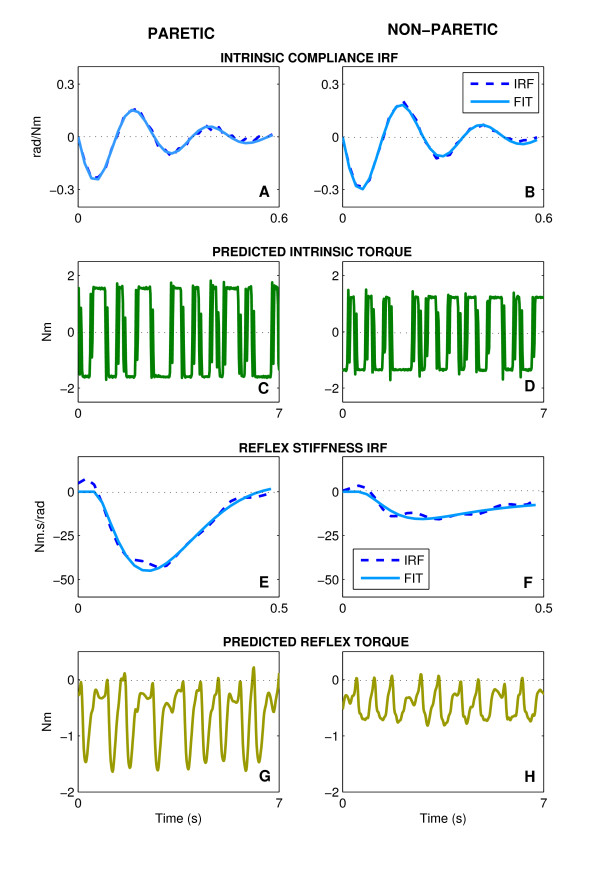
Typical intrinsic and reflex dynamics and their predicted torques estimated for the Paretic (left column) and Non-paretic (right column). **A, B **Intrinsic compliances; **C, D **Predicted intrinsic torques; **E, F **Reflex stifnness; and **G, H **Predicted reflex torques. The dashed curves are the nonparametric IRF, the solid curve are the parametric fits.

Figure 4 summarizes the intrinsic and reflex stiffness analysis for both paretic and non-paretic sides of a typical stroke subject at the NP. The dashed curves in the first row are the intrinsic compliance impulse response functions (IRFs) estimated for the paretic (Fig. 4A) and non-paretic (Fig. 4B) ankle. These were similar in shape although compliance magnitude was slightly smaller in the paretic that the non-paretic side indicating that stiffness (the inverse of compliance) was slightly larger in the paretic ankles. Second-order fits to these compliance IRFs, shown by the superimposed solid curves, were very good. In both cases, the Variance Accounted For (*VAF*_*FIT*_) was greater than 98%, as was typical of all our data; *VAF*_*FIT *_for the compliance IRF was always greater than 90%. The intrinsic torques predicted by these IRFs, shown in the Fig. 4C and 4D, were comparable in waveform although the magnitude was slightly larger in the paretic than in the non-paretic ankle, consistent with the differences in the compliance IRFs. The small differences were expected since these data were collected in the NP, at which typically there was no significant difference in the intrinsic stiffness between both sides.

The reflex stiffness IRFs, estimated from the paretic (Fig. 4E) and non-paretic (Fig. 4F) sides, are shown as dashed lines. Third-order model fits to these reflex stiffness IRFs were also very good as indicated by the superimposed solid curves. These fits were always accurate; in this case, *VAF*_*FIT *_was greater than 88% of the variance. The amplitude of reflex stiffness IRF for the paretic side (Fig. 4E) was approximately three times that of the non-paretic side (Fig. 4F). The reflex torques predicted by these IRFs shows that the peak-to-peak torque of the paretic limb in Fig. 4G (~1.5 Nm) was approximately three times that of the non-paretic limb in Fig. 4H (~0.5 Nm).

### Group data: intrinsic and reflex stiffness

**Figure 5:**
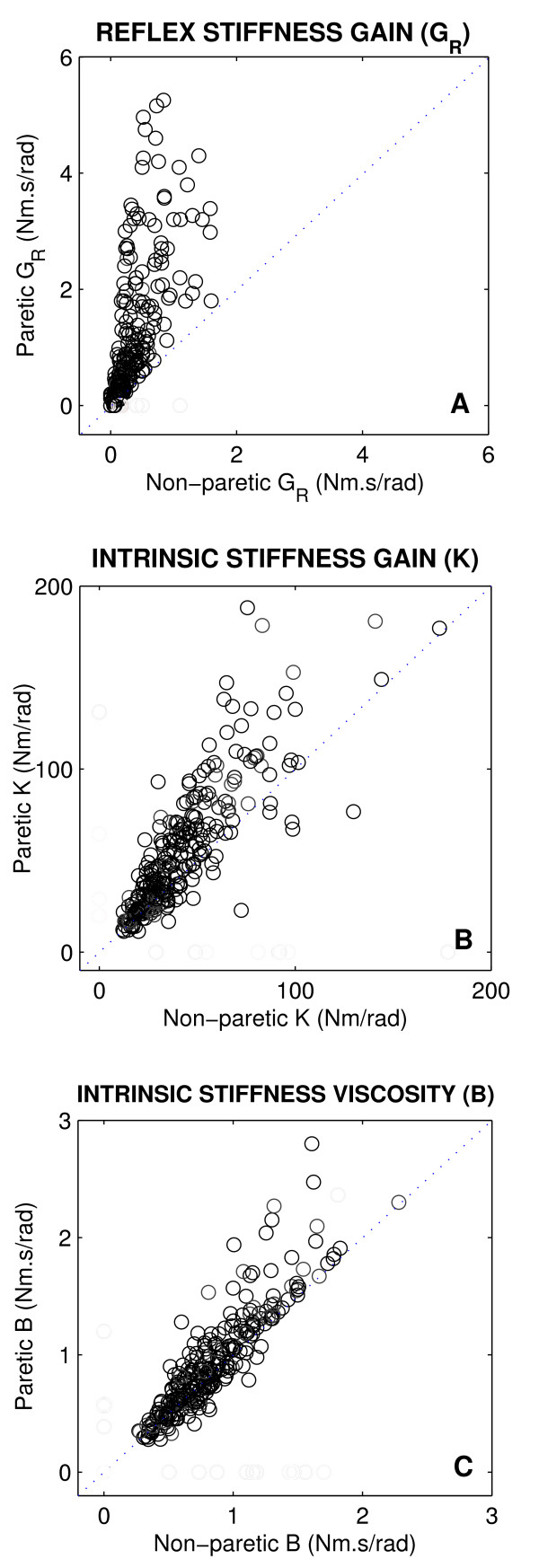
Paretic stiffness parameters plotted against non-paretic values for all stroke subjects. **A **Reflex stiffness gain (*G*_*R*_), **B **Intrinsic stiffness elasticity or gain (*K*), and **C **Intrinsic stiffness viscosity (*B*).

Figure 5 shows the intrinsic and reflex stiffness parameters from the paretic limb plotted against the corresponding control values from the non-paretic side for all stroke subjects, and for all positions. The dotted line at 45 degrees (the unity line) in each panel indicates what would be expected if there were no change due to stroke. Points above the line indicate abnormal increases following stroke, while points below the line indicate decreases.

The reflex stiffness gain values (*G*_*R*_, panel A) for all subjects were located well above the diagonal line, indicating that *G*_*R *_was larger in the paretic than in non-paretic limbs of the subjects. *G*_*R *_was the only reflex parameter that changed consistently; it increased significantly for most stroke subjects (p < 0.0001). The other three reflex parameters did not change significantly.

Similarly, the intrinsic stiffness gain (*K*, panel B) was substantially larger for the majority of stroke subjects (p < 0.0023). In contrast, the points for the intrinsic viscous parameter (*B*, panel C) were mostly clustered around the unity line, and did not show significant differences between paretic and non-paretic limbs.

### Position-dependency

**Figure 6:**
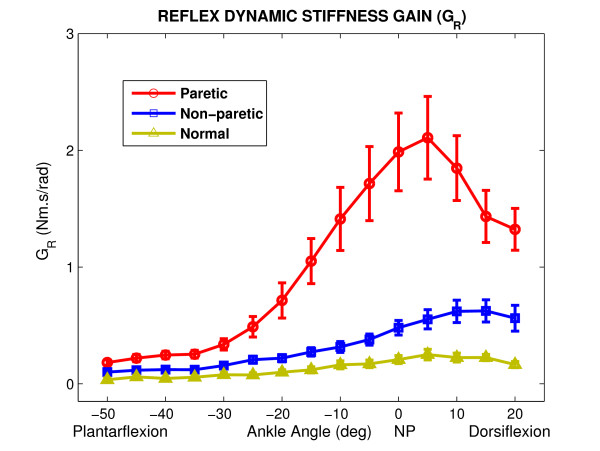
Position dependence of Reflex stiffness gain (*G*_*R*_) for paretic, non-paretic and normal groups as functions of position (Group averages). Error bars indicate ± 1 standard error. NP: Neutral Position (90°).

Figure 6 shows group average results for reflex stiffness gain as a function of ankle position for paretic, non-paretic and normal groups. There was a significant difference between the paretic group, as compared with both non-paretic and normal groups (p < 0.0001). Tukey post-hoc comparisons showed that *G*_*R *_was significantly larger in the paretic ankle than in the normal ankle at all positions (p < 0.005) and it was larger than the non-paretic ankle at all positions except for the position -50° PF (p < 0.02). Differences in *G*_*R *_increased as the ankle was dorsiflexed. Statistical analyses confirmed that there was a significant effect due to position for all groups (p < 0.0001). Position dependence was similar in all groups; the reflex stiffness gain first increased from mid-PF to mid-DF and then declined. The slope of changes was larger in the paretic than in the non-paretic and normal (P < 0.0001) groups. Similarly, the intercept of the plots relating reflex stiffness to jojnt angle increased significantly in the paretic ankle as compared to other groups (p < 0.0001).

The peak value of *G*_*R *_was around NP in the stroke ankle whereas it was around full-DF in the non-paretic and normal ankle. The group behavior was consistent but the inter-subject variability was high at mid-ROM in the stroke group as demonstrated by the large standard error bars associated with the means.

As expected from the literature [29], the non-paretic side of stroke survivors was not similar to healthy subjects; *G*_*R*_, was significantly larger in the non-paretic than the normal ankle (p < 0.001) and the differences were significant at most positions; i.e. positions between -25° PF and 20° DF, (p < 0.036).

**Figure 7:**
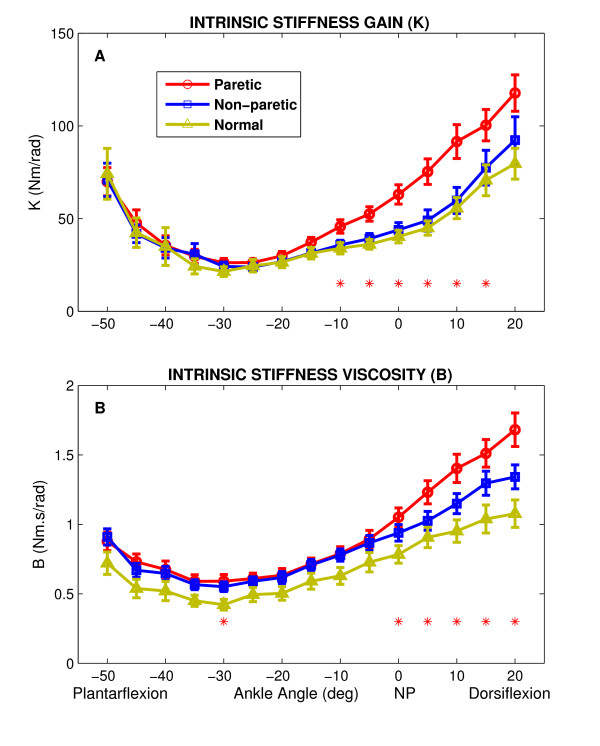
Position dependence of intrinsic stiffness for paretic, non-paretic and normal groups as functions of position (Group averages). **A **Intrinsic stiffness gain (*K*); asterisks represent points where differences between paretic group and both non-paretic and normal control groups are statistically significant. **B **Intrinsic stiffness viscous parameter (*B*); asterisks represent points where differences between paretic group and normal control group was significant. Error bars indicate ± 1 standard error. NP: Neutral Position (90°).

Figure 7 summarizes the behavior of intrinsic stiffness parameters with changes in ankle joint angle for all groups (paretic, non-paretic and normal). Overall, the group behavior was very consistent, as demonstrated by the narrow standard error bars.

For the intrinsic stiffness gain (*K*, top panel), there was a significant difference between the paretic group and both non-paretic and normal groups. *K *was significantly larger in the paretic than the non-paretic (p < 0.038) and normal (p < 0.03) ankle at dorsiflexed positions; i.e. at positions between -10° PF and 15° DF. However, the intrinsic viscous parameter (*B*, bottom panel) was significantly larger in the paretic than in the normal subjects just for positions between NP and 20° DF (p < 0.05).

Both *K *and *B *were strongly position dependent as confirmed by the statistical analysis (p < 0.0001); they first decreased sharply from full PF to mid-PF, then increased slowly from mid-PF to mid-DF, and finally it increased sharply from mid-DF to full-DF. This position dependency was consistent in all groups and was similar to our previous finding for SCI subjects [11,25].

Intrinsic stiffness gain was similar in both non-paretic and normal group (Fig. 7A) whereas the intrinsic viscous parameter increased in the non-paretic group and was significantly larger for a few positions, particularly in full DF (i.e., at 15° and 20° DF) (p < 0.05) (Fig. 7B).

### Group results: stroke effects

We investigated the position-dependency of stroke effects; i.e. the differences between paretic and non-paretic sides as ankle angle were changed systematically.

**Figure 8:**
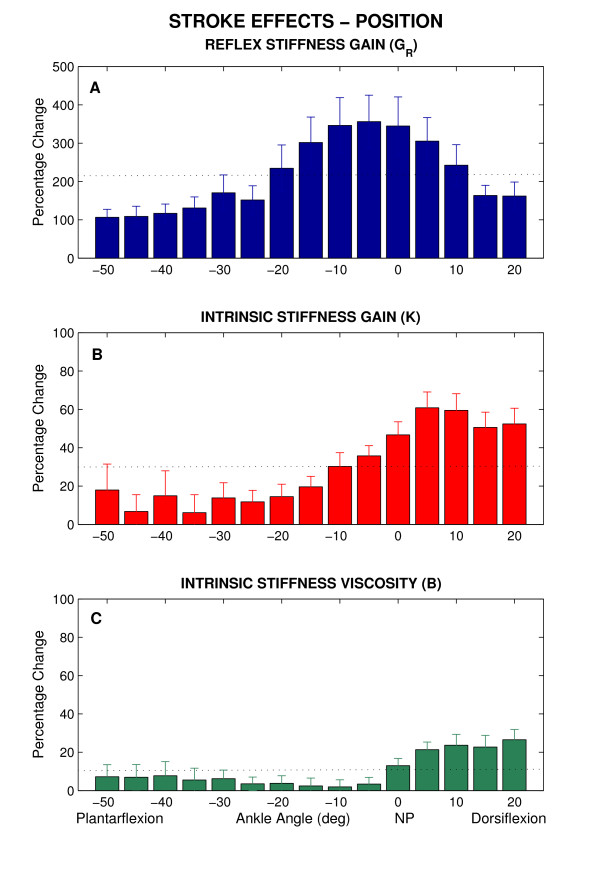
Percentage change of stroke effects as functions of position (Group averages). **A **Reflex stiffness gain (*G*_*R*_), **B **Intrinsic stiffness gain (*K*), **C **Intrinsic viscous parameter (*B*). Error bars indicate ± 1 standard error. NP: Neutral Position (90°). The dotted lines reflect the mean percentage change over the range of motion.

To characterize the amplitude of these changes, we computed the percentage change caused by stroke (stroke effects) at each joint position in stroke patients. Figure 8 shows the changes in *G*_*R*_*, K*, and *B *and as a function of position. Panel A shows that *G*_*R*_, increased in stroke subjects between ~100% at full-PF and ~350% around NP, by an average of 211 ± 92%. The highest percentages of changes obtained from mid-ROM. Panel B and C show that *K *and *B *also increased by an average of 30 ± 19% and 10 ± 8%, respectively, which are much smaller than the percentage of increase in reflex stiffness gain. However, an increase of ~50% was observed for *K *only at dorsiflexed positions which was considerable. These changes clearly indicate that the abnormalities in intrinsic and reflex stiffness are strongly position dependent.

## Discussion

Our results revealed that both neural and muscular systems are altered in spastic limbs, but the changes are complex and may depend on several factors. In this study, we probed changes in intrinsic stiffness and changes in reflex stiffness as a function of joint angle over the entire angular range of motion, and found strong position dependency in these neuromuscular abnormalities.

### Summary of results

We used the parallel cascade system identification technique to characterize the mechanical changes associated with spasticity in the ankle joint of chronic hemiplegic stroke subjects. To our knowledge, this is the first study that quantified the changes in neuromuscular properties over the entire ROM, and used two different control groups; i.e. the non-paretic limb in the stroke patients and the normal limb in the healthy subjects.

Our major findings were that,

(i) overall dynamic joint stiffness was increased in paretic side,

(ii) both reflex and intrinsic stiffness gain was larger in paretic than in the non-paretic and normal limb and contributed substantially to the increased stiffness,

(iii) these abnormalities were strongly dependent on joint position; reflex stiffness was most pronounced at mid-ROM whereas intrinsic stiffness were dominant during DF,

(iv) the non-paretic side of people with stroke was not similar to that of healthy ankle muscles in control subjects. Reflex stiffness gain was significantly larger in them than in healthy ankle muscles. Intrinsic viscosity was also larger in the non-paretic than in the normal side but the differences were not significant.

### Increased intrinsic stiffness

We found that the intrinsic stiffness and viscous parameter were larger in the stroke than in the normal subjects (Figure 7), and the differences were significant for DF. Increased intrinsic stiffness is consistent with enhancement in passive stiffness of the ankle joint reported by Sinkjaer et al. [12]. Surprisingly, Galiana et al. found no significant differences between these groups [26]. This discrepancy can be explained by two major differences between the two studies.

First, Galiana et al. [26] studied a limited range of positions; e.g. from mid-PF to NP position, where the differences between intrinsic stiffness of stroke and normal subjects were small, according to our findings. This emphasizes the importance of considering the position dependency of joint dynamic stiffness and its intrinsic and reflex components. Second, the time post-injury which can play a critical role in developing intrinsic structural remodeling was different between two studies; the average time post-lesion used in their study (approximately 10.5 months) was much shorter than that in our studies (approximately 92 months). Thus, lack of changes in intrinsic stiffness observed by Galiana et al. [26] could be due to shorter post-lesion times in their patients which were potentially not long enough for the development of substantial muscle fiber remodeling.

Recent cellular studies may explain the enhanced intrinsic stiffness we observed in our stroke subjects with chronic spasticity. Published studies of the tensile modulus of muscle fibers demonstrated that intrinsic stiffness of spastic muscle fibers is increased [33,34]. Furthermore, the resting sarcomere length of cells is shorter in spastic muscle cells [35,36]. Finally, although it has been proposed that the isoform of titin, a large intracellular cytoskeletal protein, may also be altered in spastic muscles and contribute to these changes [33,37], recent findings reveal no change in titin isoforms in spastic muscle [38].

In addition to altered muscle cell properties, changes in proliferation of extracellar matrix material and in the mechanical properties of this extracellular material in spastic muscle are described, based on biochemical measurement of collagen concentration [39]. It has been suggested that although spastic muscle contains a larger amount of extracellular matrix material, the quality of that material is much different from that in normal muscles [33,35]. Taken together, the intra- and extracellular alterations in muscle and its tissue that usually occur secondary to spasticity will likely change the intrinsic mechanical properties of spastic limbs.

Intrinsic stiffness may also change for other structural reasons such as alteration in fiber size and fiber type distributions and potentially also fiber length. Micrographs of spastic muscles showed increased fiber size variability [9,40,41] which may result from muscle fiber thinning that usually occur near the end of some fibers [42,43]. The occurrence of changes in fiber type distribution secondary to spasticity is also accepted [35], although the nature of these changes is controversial [44-46]. It has been also widely reported that muscle fiber length and the number of sarcomeres within the muscle decrease secondary to spasticity, contributing to contracture [47,48]. Conversely, there is evidence indicating that serial sarcomere number can be increased by lengthening the position at which muscle immobilization occurs [49]. While patients with stroke are not fully immobilized, particularly at longer resting lengths, the lack of mobility of patients post-stroke may provide some of the immobilization stimulus. Although this adaptability to chronic length changes cannot be generalized to all muscles [35] changes in serial sarcomere number have also been reported for the ankle extensor muscles [49]. It is also known that the resting sarcomere length of spastic fibers is reduced in the spastic muscle [33,34]. These changes associated with sarcomere length reduction, and with the accumulation of connective tissues in atrophic muscles secondary to the lesions, may cause a left shift in passive length-force curves that can explain the position dependency of altered intrinsic stiffness found in the current study.

### Abnormalities in reflex stiffness

Our results demonstrate that reflex stiffness gain was significantly larger in stroke than normal subjects at most positions; in some cases by as much as a factor of seven. The differences were greatest at mid-ROM where reflex stiffness was largest for the stroke group. In contrast, others reported reflex torque [12] and stiffness [26] of spastic subjects within the normal range. This discrepancy is at least partially related to methodological differences as described below.

In the Sinkjaer study [12] the amplitude of the torque response was divided by that of the perturbation to provide units of *stiffness*, assuming that joint stiffness is a linear property that did not vary with displacement amplitude or joint angle. Since both intrinsic and reflex mechanics are known to be highly non-linear, and to be dependent on joint angle, these measurements are unlikely to provide an accurate measure of joint properties. The Sinkjaer study also attempted to separate intrinsic muscular contributions from reflex contributions using electrical stimulation of the muscle nerve. There is some uncertainty in interpreting experiments using electrical stimulation to assess muscle mechanical properties [12,16,18], since the order of motor axon recruitment during electrical stimulation is very different from that arising during normal voluntary contractions [50,51]. Thus, reflex contributions cannot be accurately estimated by these methods.

Our results are at variance with those of Galiana et al. [26]. This difference could be due to their smaller sample size, particularly since they report substantial increases in the reflex response in four out of eleven patients.

Our findings demonstrate that reflex stiffness gain was strongly dependent on position, similar to our previous findings in subjects with SCI [11,25]. Indeed, reflex stiffness gain was modulated greatly with ankle position; it increased from PF to mid-DF in both stroke and normal groups but it declined in the stroke subjects as the ankle was moved toward full-DF. This abnormal modulation at DF was not reported in the Galiana study, because their subjects were examined over a more limited ROM, which did not reach beyond the neutral joint angle.

The increases in reflex stiffness could be attributed to greater excitation of the motoneuron pool by augmented afferent or interneuronal input. Under these conditions, recruitment of motoneurons would likely follow the normal sequence, from small to large. Alternatively, augmented reflex stiffness gain could be due to an inappropriate recruitment sequence, in the paretic limb, in which recruitment rank order is disrupted, or even reversed. This idea has been supported by others, who suggest that CNS lesions may alter motor output by inducing new connections including sprouting and strengthening of existing connections to optimize their performance [52].

Abnormal modulation of reflex stiffness gain with ankle position in the paretic limb was characterized by the three major changes: (1) the slope of the gain was greater from -30° PF to 5° DF, (2) the ordinal intercept was larger, and (3) the reflex gain decreased sharply at the end of DF.

The first two changes can be explained by enhancement of reflex gain as explained above. The decline in the reflex gain at the end of DF, however, could be due potentially to inhibitory effects of group III/IV muscle afferents which are activated preferentially as muscles are stretched to near maximum length [53]. The increased tension, which may happen at full joint DF in normal subjects, was present at the narrower joint angle in the spastic joints due to the existence of muscle hypertonia. This abnormal modulation can elicit strong reflex responses at undesirable posture and/or phase during movement, and consequently contribute to movement impairments as observed in the transition from stance to swing during walking in spastic patients [54].

### Non-paretic limb of stroke patients versus normal limb

Since the non-paretic limb of the stroke patients has initially similar mass, muscle architecture and neural connectivity to the paretic limb, it seems that it could be the best control because it reduces the inter-subject variability. In contrast, it has been suggested that the non-paretic limb in both the upper [55,56] and lower extremities [57-59] is influenced to some extent by stroke. However, the comparison between neuromechanical properties of the normal limb in healthy subjects and non-paretic limb in persons with stroke has not been done yet. We postulated that the non-paretic ankle would have different neuromuscular properties than the normal ankle. We therefore probed this hypothesis by comparing intrinsic viscoelastic parameters and reflex stiffness gain between stroke patients and aged-matched healthy subjects. Our results revealed that all these parameters were larger in the non-paretic than in the healthy subjects' ankle but the differences were significant only for reflex stiffness. These findings suggest that the non-paretic lower extremity of people with stroke may not be used as an appropriate control for the study of neuromuscular properties.

### Significance

The abnormalities in spastic, paretic joint stiffness, and its intrinsic and reflex components, vary systematically with position over the full angular range of motion. When the ankle was fully plantarflexed, there was no systematic difference between the overall joint stiffness of spastic and normal joints. In the mid range, overall joint stiffness was greater in spastic limbs primarily due to abnormally high reflex stiffness. At full DF, increased intrinsic stiffness provided the major contribution to the larger overall stiffness in spastic joints. Thus, questions about the nature and origin of hypertonia may have quite different answers depending upon where in the ROM tests are made. This can explain some controversies in the literature regarding the nature and origins of mechanical abnormalities associated with spasticity.

## Conclusion

Our findings revealed that in the paretic ankle of hemiparetic stroke survivors, both intrinsic and reflex stiffnesses were significantly increased, as compared to the non-paretic ankle joints of stroke survivors, and to normal controls. These abnormalities varied systematically and differently with changing ankle position, over the full angular range of motion; the differences reached their maximum at NP for the reflex stiffness and at DF for the intrinsic stiffness. These findings indicate that clinical perceptions of increased tone may have quite different origins depending upon the angle where the tests are initiated.

Furthermore, reflex stiffness was significantly larger in the non-paretic limb of stroke patients than in healthy control subjects, suggesting that the non-paretic limb should not be used as control for studying neuromuscular properties of the ankle joint.

Our findings will help elucidate the origins of the neuromuscular abnormalities associated with stroke-induced spasticity and may facilitate the development of targeted interventions for preventing these abnormal changes or reversing them.

## Abbreviations

ROM: Range of Motion; MAS: Modified Ashworth Scale; DF: Dorsiflexion; PF: Platarflexion; EMGs: Electormygrams; GS: Gastrocnemius; TA: Tibiliais anterior; PRBS: Pseudorandom Binary Sequence; IRF: Impulse Response Function; VAF: Variance Accounted for; G_R:_ Reflex stiffness gain; K: Intrinsic stiffness gain; B: Intrinsic stiffness viscosity; NP: Neutral Position.

## Competing interests

The authors declare that they have no competing interests.

## Authors' contributions

MMM designed the study, supervised data collection and analysis, and participated in interpreting and writing the manuscript. LA participated in performing the experiments, interpreting data and writing the paper, MT participated in analyzing data, and WZR participated in interpreting data and writing the manuscript. All authors read and approved the final manuscript.
